# Adolescent Female Cannabinoid Exposure Diminishes the Reward-Facilitating Effects of Δ^9^-Tetrahydrocannabinol and *d*-Amphetamine in the Adult Male Offspring

**DOI:** 10.3389/fphar.2017.00225

**Published:** 2017-04-25

**Authors:** George Pitsilis, Dimitrios Spyridakos, George G. Nomikos, George Panagis

**Affiliations:** ^1^Laboratory of Behavioral Neuroscience, Department of Psychology, School of Social Science, University of CreteRethymno, Greece; ^2^Global Medical Science, Sage Therapeutics, CambridgeMA, USA

**Keywords:** cannabis, cannabinoid, *d*-amphetamine, psychostimulants, intracranial self-stimulation, adolescence, cross-generational

## Abstract

Marijuana is currently the most commonly abused illicit drug. According to recent studies, cannabinoid use occurring prior to pregnancy can impact brain plasticity and behavior in future generations. The purpose of the present study was to determine whether adolescent exposure of female rats to Δ^9^-tetrahydrocannabinol (Δ^9^-THC) induces transgenerational effects on the reward-facilitating effects of Δ^9^-THC and *d*-amphetamine in their adult male offspring. Female Sprague-Dawley rats received Δ^9^-THC (0.1 or 1 mg/kg, i.p.) or vehicle during postnatal days 28–50. As adults, females were mated with drug-naïve males. We then assessed potential alterations of the Δ^9^-THC’s (0, 0.1, 0.5, and 1 mg/kg, i.p.) and *d*-amphetamine’s (0, 0.1, 0.5, and 1 mg/kg, i.p.) reward-modifying effects using the curve-shift variant of the intracranial self-stimulation (ICSS) procedure in their adult male F1 offspring. The reward-facilitating effect of the 0.1 mg dose of Δ^9^-THC was abolished in the F1 offspring of females that were exposed to Δ^9^-THC (0.1 or 1 mg/kg), whereas the reward-attenuating effect of the 1 mg dose of Δ^9^-THC remained unaltered. The reward-facilitating effects of 0.5 and 1 mg of *d*-amphetamine were significantly decreased in the F1 offspring of females that were exposed to Δ^9^-THC (1 mg/kg and 0.1 or 1 mg, respectively). The present results reveal that female Δ^9^-THC exposure during adolescence can diminish the reward-facilitating effects of Δ^9^-THC and *d*-amphetamine in the adult male offspring. These transgenerational effects occur in the absence of *in utero* exposure. It is speculated that Δ^9^-THC exposure during female adolescence may affect neural mechanisms that are shaping reward-related behavioral responses in a subsequent generation, as indicated by the shifts in the reward-facilitating effects of commonly used and abused drugs.

## Introduction

Marijuana is the most widely abused illicit drug in the world with use often initiated during adolescence. One additional concern is the high percentage of adolescents that use marijuana and the increase in use among female adolescents, as reported over the last few years (Center for Behavioral Health Statistics, and Quality, 2016). Marijuana use among adolescents may be particularly problematic since this period is associated with the maturation of the endocannabinoid system in brain regions implicated in reward and motivation (Marco and Laviola, 2012). Thus, individuals in this period may be particularly vulnerable to the long-term effects of marijuana. Although a number of preclinical studies have examined the impact of cannabinoid administration during adolescence on the adult brain structure and function (Viveros et al., 2005; Campolongo et al., 2011), only a limited number of studies have examined the consequences of cannabinoid exposure during this distinct developmental period that can be passed down to future generations (Vassoler et al., 2014; Yohn et al., 2015; Szutorisz and Hurd, 2016).

Interestingly, recent studies point out that parental and/or maternal cannabinoid exposure, occurred in prereproductive phase, can affect neural and behavioral development of future offspring. Indeed, male offspring of female rats treated during their adolescence with the CB1 receptor agonist WIN55,212-2 exhibited enhanced morphine-induced conditioned place preference (Byrnes et al., 2012). Similarly, female offspring of female rats treated during their adolescence with WIN55,212-2 demonstrated enhanced expression of morphine-induced locomotor sensitization (Byrnes et al., 2012). These findings support the hypothesis that adolescent female cannabinoid exposure induces transgenerational behavioral effects in both male and female offspring in the absence of *in utero* exposure. Notably, in a more recent study, parental germline Δ^9^-THC exposure during adolescence resulted in increased heroin self-administration in adult male offspring; moreover, offspring displayed altered stereotyped and approach-avoidance behaviors during the period of acute heroin withdrawal (Szutorisz et al., 2014). On a molecular level, these effects have been associated with changes in the mRNA expression of cannabinoid, dopamine, and glutamatergic receptor genes in the striatum, a brain region implicated in reward, compulsive behaviors and drug addiction (Szutorisz et al., 2014). Interestingly, in a later study, DNA methylation disturbances were detected in the nucleus accumbens of adult rats with parental germline Δ^9^-THC exposure (Watson et al., 2015).

Several studies proposed a closed interaction between the endocannabinoid and the opioid systems (Robledo et al., 2008; Parolaro et al., 2010). Accordingly, to date studies have only examined the consequence of cannabinoid exposure on opioid responses in offspring. Thus, the aim of the present study was to investigate alterations in the well-documented reward-modifying effects of Δ^9^-THC and *d*-amphetamine in the adult male offspring due to adolescent mother exposure to Δ^9^-THC. To this end, we treated female adolescent rats with two different doses of Δ^9^-THC, a low dose that acutely induces rewarding and a high dose that acutely induces anhedonic effects in the intracranial self-stimulation paradigm (ICSS) paradigm in drug-naïve rats, according to previous studies from our laboratory (Katsidoni et al., 2013); following mating with drug-naïve male rats, the reward-modifying effects of Δ^9^-THC and *d*-amphetamine were assessed in their adult male offspring.

## Materials and Methods

### Animals

Sprague-Dawley rats were used. Animals were group-housed in standard plastic laboratory cages under a 12 h dark- 12 h light cycle (lights on at 08.00 a.m, lights off at 20.00 p.m), with food and water available *ad libitum*. Procedures and testing were performed during the light phase. All the experiments were conducted in accordance with the European Communities Council Directive (86/609/EEC) and the National Institutes of Health *Guide for the Care and Use of Laboratory Animals* and were approved by the Experimental Animal Ethics Committee of the Department of Psychology, University of Crete. All efforts were made to minimize animal suffering and to reduce the number of animals used.

### Drugs and Administrations

Δ^9^-THC was dissolved in a vehicle solution which was consisted of 5% DMSO (dimethylsulfoxide), 5% cremophor and 90% saline (sodium chloride solution 0.9%) and was administrated in a volume of 3 ml/kg of body weight intraperitoneally (i.p.). *d*-Amphetamine hydrochloride was dissolved in a saline solution (sodium chloride solution 0.9%) and was administrated in a volume of 1 ml/kg of body weight intraperitoneally (i.p.). The doses used in the present study were based on previous studies from our laboratory (Vlachou et al., 2007; Mavrikaki et al., 2010; Katsidoni et al., 2013).

### Cross-Generational Δ^9^-THC Animal Model (F0 Female Animals and F1 Male Animals)

The animals used in the present study were the female littermates bred in our laboratory. From postnatal day 25 to postnatal day 27, female animals were being familiarized with the researchers to eliminate any stress effect later during the experimental procedures. Briefly, female rats were exposed to either Δ^9^-THC (0.1 and 1 mg/kg, i.p.) or vehicle, one injection every 2nd day during postnatal days 28–50 (12 injections in total). Thus, female animals have been divided into three separate groups based on the treatment received: females exposed to 0.1 mg/kg of Δ^9^-THC (F0-Δ^9^-THC 0.1), females exposed to 1 mg/kg of Δ^9^-THC (F0-Δ^9^-THC 1) and females exposed to vehicle (F0-VEH). Fifteen to nineteen days after the final drug or vehicle administration (postnatal days 65 to 69), all females were mated with drug naïve colony males in a ratio of two females to one male. Prior to parturition females were separated and single housed. After birth, litters were culled to 8–10 pups. All male offspring were weaned and group-housed (3–4 rats/cage) on postnatal day 21 and remained undisturbed until the time of surgery. Cages were assigned to the same experimental group and three experimental groups of F1 male animals were set up: F1 animals which their mothers that had been exposed to the vehicle of Δ^9^-THC (F1 of F0-VEH), F1 animals which their mothers had been exposed to the dose 0.1 mg/kg of Δ^9^-THC (F1 of F0-Δ^9^-THC0.1) and F1 animals which their mothers had been exposed to the dose 1 mg/kg of Δ^9^-THC (F1 of F0-Δ^9^-THC1). All F1 animals were then assessed during their adulthood for potential alterations in the reward-facilitating effects of either Δ^9^-THC or *d*-amphetamine using the curve-shift variant of the ICSS procedure.

No long-term, differentiating effects of the adolescent exposure to Δ^9^-THC compared to placebo on the behavior or other physiological parameters (e.g., gross locomotor activity, reaction to handling, social interaction, self-grooming, body weight) of the female rats, as adolescents or mothers, or their adult male offspring were observed during the study.

### Stereotaxic Surgery and ICSS Procedures

At postnatal day 90, F1 animals were stereotaxically implanted with a monopolar stimulation electrode, as previously described (Katsidoni et al., 2011). After 1 week recovery, the F1 rats were tested for self-stimulation in an operant chamber made of transparent Plexiglas (25-cm wide, 25-cm deep, and 30-cm high). Each chamber was equipped with a stainless-steel poke device (lever) 4-cm wide and protruded 2 cm from the left side at a height of 4 cm from the bottom. Each bar-press triggered a constant current stimulator (Med Associates, St. Albans, VT, USA) that delivered a 0.4-s train of rectangular cathodal pulses of constant duration (0.1 ms) and intensity (250 μA) and variable frequency (15–100 Hz, i.e., 6–40 number of pulses/0.4 s) increased up to 40 per stimulation train until the animal/rat showed vigorous self-stimulation. During the acquisition phase, the animals were trained to self-stimulate for at least three consecutive days (1 h daily), using stimulation parameters that maintained near maximal bar-pressing rates. After self-stimulation was acquired and stabilized for a given pulse frequency, rats were trained to self-stimulate using four alternating series of ascending and descending pulse frequencies. The pulse frequency was varied by steps of approximately 0.1 log units. Each frequency was tested within trials of 60 s in duration, followed by an extinction period of 30 s. For each trial, there was an initial “priming” phase during which the animals received three trains of stimulation at the frequency which was available for the specific trial. A rate–frequency determination session lasted approximately 45 min. One rate–frequency curve was established daily, for 10–14 days, depending on the period when the self-stimulation indices (i.e., curve shift and threshold measure) were stable; the stabilization period of this parameter varies between rats, but curves remain stable, when established in each rat. The stimulation parameters, ICSS sessions, and data collection were controlled by a computer. Drug testing (with Δ^9^-THC or *d*-amphetamine) began for each animal when the function relating bar-pressing rate to pulse frequency (the rate-frequency function) was stable for at least three consecutive days. The criterion for stability was met when the frequency thresholds did not vary by more than 0.1 log units for at least three consecutive days.

### ICSS Experiments

#### Experiment 1: Effects of Δ^9^-THC on Brain Stimulation Reward of F1 Animals

The aim of the first experiment was to examine in the three groups of F1 animals [i.e., F1 of F0-VEH (*n* = 8), F1 of F0-Δ^9^-THC0.1 (*n* = 8) and F1 of F0-Δ^9^-THC1 (*n* = 8)] the effects of the acute systemic administration of Δ^9^-THC on brain stimulation reward. Rats received multiple doses of Δ^9^-THC (0, 0.1, 0.5, and 1 mg/kg, i.p.). The sequence of injections for the different drug doses was counterbalanced with respect to order and a 3-day period was allowed between injections. The drug or vehicle self-stimulation test consisted of a pre-drug and two post-drug rate-frequency function determinations (for 45 min each). Δ^9^-THC was administered immediately following the pre-drug rate–frequency function determination. The first session began 20 min postinjection, while the second session started 70 min after Δ^9^-THC injection.

#### Experiment 2: Effects of *d*-Amphetamine on Brain Stimulation Reward of F1 Animals

The aim of the second experiment was to examine in a different set of three groups of F1 animals (i.e., F1 of F0-VEH (*n* = 8), F1 of F0-Δ^9^-THC0.1 (*n* = 8) and F1 of F0-Δ^9^-THC1 (*n* = 8)] the effects of the acute systemic administration of *d*-amphetamine on brain stimulation reward. Rats received multiple doses of *d*-amphetamine (0, 0.1, 0.5, and 1 mg/kg, i.p.). The sequence of injections for the different drug doses was counterbalanced with respect to order and a 3-day period was allowed between injections. The drug or vehicle self-stimulation test consisted of a pre-drug and a post-drug rate-frequency function determination (for 45 min each). *d*-Amphetamine was administered immediately following the pre-drug rate–frequency function determination. After a postinjection interval of 5 min, the rats were placed in the operant chamber, and the post-drug session began.

### Data and Statistical Analysis

The analysis was performed on two aspects of data obtained from the rate-frequency curve: the ICSS threshold and the maximum rate of responding or asymptote, as it has previously been described (Katsidoni et al., 2011). The post-treatment threshold and asymptote values were expressed as percentage of pre-treatment values. In the first experiment, the significance of the drug effect and time was statistically evaluated initially using one-way analysis of variance (ANOVA). When one-way analysis of variance had a statistically significant effect, we used independent sample *t-*test to compare the means among the three groups. Also in order to compare the difference between first and second postinjection session within each of three groups, we conducted paired sample *t*-test. In the second experiment, the significance of the drug effect was statistically evaluated initially using one-way analysis of variance (ANOVA). Statistically significant results in ANOVA were followed by independent sample *t*-test and the analysis of simple effects was tested in a:

$$p=\frac{\text{The sum of ps for the main plus interaction effects}}{\text{Number of simple effects}}$$

The significance of effects of Δ^9^-THC (0, 0.1, 0.5, and 1 mg/kg) and *d*-amphetamine (0, 0.1, 0.5, and 1 mg/kg) in the threshold of F0-VEH group, at the two separate experimental settings, outlined above as Experiment 1 and Experiment 2 respectively, was evaluated using two-way analysis of variance (ANOVA) with repeated measures, followed whenever appropriate, by correlated *t*-test using Bonferroni’s adjustment for multiple comparisons. The level of significance was set at 0.05. Statistical analyses were conducted using the Statistical Package for the Social Sciences v.20.0 (SPSS, Chicago, IL, USA).

## Results

### Experiment 1: Effects of Δ^9^-THC on Brain Stimulation Reward of F1 Animals

**FIGURE 1:**
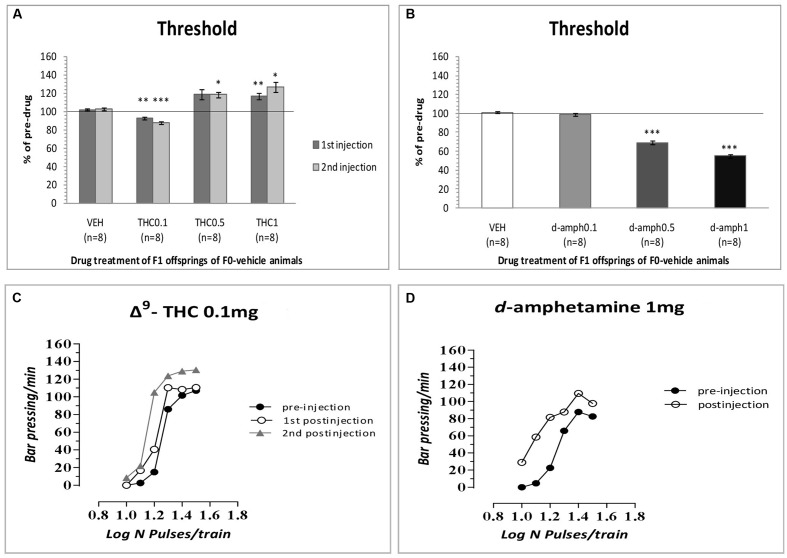
**Changes in intracranial self-stimulation (ICSS) threshold expressed as percentage of predrug values, following Δ^9^-THC (0, 0.1, 0.5, and 1 mg/kg, i.p.)** **(A)** and *d*-amphetamine (0, 0.1, 0.5, and 1 mg/kg, i.p.) **(B)** administration in F1 offspring of F0-vehicle animals. Vertical bars represent the means ± SEM. The asterisk (^∗^) signifies an ICSS threshold significantly different from the respective control group (vehicle), ^∗^*p* < 0.05, ^∗∗^*p* < 0.01, ^∗∗∗^*p* < 0.001. **(C,D)**: Rate-frequency functions (rate of lever pressing as a function of stimulation frequency) taken from representative drug naïve animals (i.e., F1 of F0-VEH animals). Each plot represents data from a single animal under predrug and drug conditions (**C**: Δ^9^-THC0.1 mg/kg, **D**: *d*-amphetamine 1 mg/kg). Rate frequency functions were obtained by logarithmically decreasing the frequency of the stimulation pulses from a value that sustained maximal lever pressing to one that failed to sustain lever pressing. Both Δ^9^-THC and *d*-amphetamine caused parallel leftward shifts in the rate-frequency function.

The results of the first experiment replicated our previous findings that Δ^9^-THC at the dose of 0.1 mg/kg increased the rewarding efficacy of self-stimulation in drug naïve animals, i.e., F1 of F0-VEH animals (see **Figure 1A**) and caused leftward shifts in the rate-frequency function (see **Figure 1C**). Specifically, two-way ANOVA with repeated measures showed a statistically significant interaction of Δ^9^-THC and time [*F*(3,21) = 17.838, *p* = 0.001] on the ICSS threshold. Repeated measures on the simple effect of 1st postinjection showed a statistical significant effect of Δ^9^-THC [*F*(1,7) = 37.602, *p* = 0.001]. Paired-sample *t*-test using Bonferroni’s adjustment for multiple comparisons revealed that Δ^9^-THC at the dose of 0.1 mg/kg significantly decreased (*p* = 0.003), while at the dose of 1 mg/kg significantly increased the ICSS threshold (*p* = 0.010). The dose of 0.5 mg/kg did not produce any significant effect (*p* = 0.057). Repeated measures on the simple effect of 2nd postinjection showed a statistically significant effect of Δ^9^-THC [*F*(1,7) = 24.846, *p* = 0.0001]. Paired-sample *t*-test using Bonferroni’s adjustment for multiple comparisons revealed that Δ^9^-THC at the dose of 0.1 mg/kg significantly decreased (*p* = 0.001), while at the dose of 1 mg/kg significantly increased the ICSS threshold (*p* = 0.020). Besides, the dose of 0.5 mg/kg significantly increased the ICSS threshold (*p* = 0.012).

**FIGURE 2:**
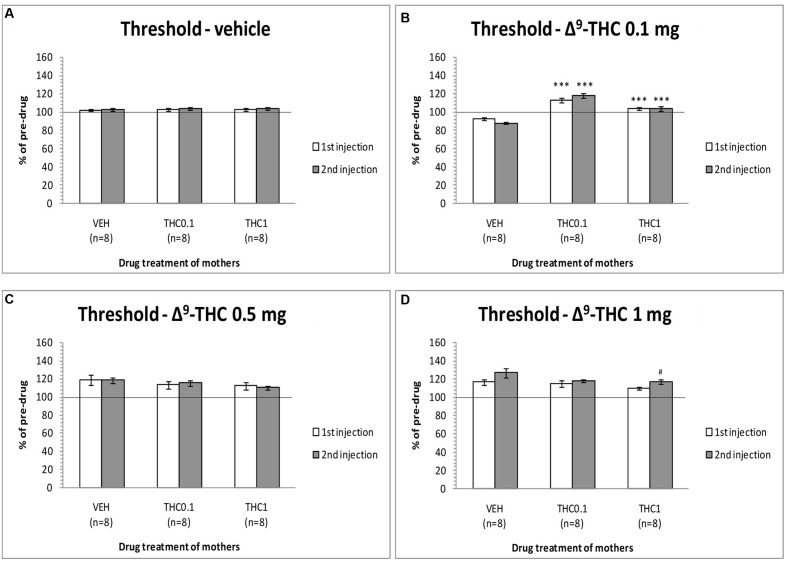
**Changes in self-stimulation threshold in F1 animals, expressed as percentage of predrug value, following acute i.p. administration of 0 mg/kg** **(A)**, 0.1 mg/kg **(B)**, 0.5 mg/kg **(C)**, and 1 mg/kg **(D)** of Δ^9^-THC, during first and second postinjection session. Horizontal axes represent the three groups of F1 animals. The *asterisk* (^∗^) signifies a statistically significant effect compared to the vehicle group (F1 of F0-VEH animals), ^∗∗∗^*p* < 0.001. ^#^signifies a statistically significant effect compared to the first postinjection of the same group, ^#^*p* < 0.05.

The changes in ICSS threshold of F1 animals after acute systemic injection of Δ^9^-THC or its vehicle are presented in **Figure 2**. One-way ANOVA showed that the dose 0 mg/kg (namely the vehicle of Δ^9^-THC), did not affect the threshold of F1 of F0-Δ^9^-THC0.1 animals and F1 of F0-Δ^9^-THC1 animals compared to F0-VEH animals, neither at the first postinjection session [*F*(2,21) = 0.015, *p* = 0.985] nor at the second postinjection session [*F*(2,21) = 0.404, *p* = 0.673]. Paired sample *t*-test for the comparison between the first postinjection and second postinjection session of F1 of F0-VEH animals (*p* = 0.924), F1 of F0-Δ^9^-THC0.1 (*p* = 0.469) and F1 of F0-Δ^9^-THC1 animals (*p* = 0.361), did not reveal any statistically significant difference. On the other hand, Δ^9^-THC at the dose 0.1 mg/kg, significantly affected the threshold of F1 animals at the first postinjection [*F*(2,21) = 26.989, *p* = 0.001]. Further analysis for the first postinjection session using independent sample *t*-test revealed that Δ^9^-THC at the dose 0.1 mg/kg increased the threshold of F1 of F0-Δ^9^-THC0.1 animals [*t*(10.214) = -6.767, *p* = 0.001] and also increased the threshold of F1 of F0-Δ^9^-THC1 animals [*t*(14) = -5.430, *p* = 0.001], compared to the control group, namely F1 of F0-VEH animals. Similarly, one-way ANOVA for the dose 0.1 mg/kg of Δ^9^-THC at the second postinjection session, revealed statistical significant effects [*F*(2,21) = 43.520, *p* = 0.001]. Independent sample *t*-test showed that Δ^9^-THC at the dose of 0.1 mg/kg increased significantly the threshold both of F1 of F0-Δ^9^-THC0.1 animals [*t*(14) = -10.054, *p* = 0.001] and F1 of F0-Δ^9^-THC1 animals [*t*(14) = -5.605, *p* = 0.001], compared to the threshold of F1 of F0-VEH animals. Furthermore paired sample *t*-test did not reveal any statistically significant difference between the first and second postinjection session in F1 of F0-VEH animals (*p* = 0.071), F1 of F0-Δ^9^-THC0.1 animals (*p* = 0.130) and F1 of F0-Δ^9^-THC1 animals (*p* = 0.853). Δ^9^-THC at the dose 0.5 mg/kg did not affect the threshold of F1 of F0-Δ^9^-THC0.1 animals and F1 of F0-Δ^9^-THC1 animals neither during the first postinjection session [*F*(2,21) = 0.657, *p* = 0.529], nor at the second postinjection session [*F*(2,21) = 1.947, *p* = 0.168]. Paired sample *t*-test did not reveal any statistically significant difference in F1 of F0-VEH (*p* = 0.927), F1 of F0-Δ^9^-THC0.1 (*p* = 0.724) and F1 of F0-Δ^9^-THC1 animals (*p* = 0.617), between the first and the second postinjection. Δ^9^-THC at the dose 1 mg/kg did not affect the threshold of both F1 of F0-Δ^9^-THC0.1 animals and of F0-Δ^9^-THC1 animals, compared to F1 of F0-VEH animals, neither at the first postinjection session [*F*(2,21) = 1.196, *p* = 0.322], nor at the second postinjection session [*F*(2,21) = 2.329, *p* = 0.122]. Paired sample *t*-test did not reveal any statistically significant difference between first and second postinjection session in F0-VEH group (*p* = 0.069) and F0-Δ^9^-THC0.1 group (*p* = 0.327), but there was a statistically significant difference in F0-Δ^9^-THC1 group [*t*(7) = -3.117, *p* = 0.017]. Rate-frequency functions (rate of lever pressing as a function of stimulation frequency) taken from representative F1 animals are presented in **Figure 6A** (mother treatment Δ^9^-THC 0.1 mg) and **Figure 6B** (mother treatment Δ^9^-THC 1 mg). These plots demonstrate that the reward-facilitating effect of Δ^9^-THC in F1 animals (vis-à-vis **Figure 1C**) has been abolished.

**FIGURE 3:**
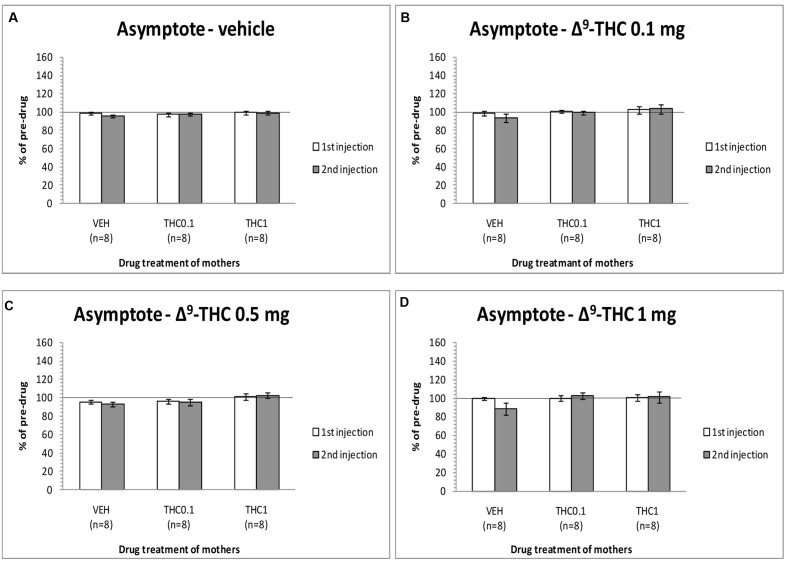
**Changes in asymptotic rate of responding in F1 animals, expressed as percentage of predrug value, following acute i.p. administration of 0 mg/kg** **(A)**, 0.1 mg/kg **(B)**, 0.5 mg/kg **(C)**, and 1 mg/kg **(D)** of Δ^9^-THC, during first and second postinjection session. Horizontal axes represent the three groups of F1 animals.

The changes in the asymptotic rate of responding of F1 animals after acute systemic injection of Δ^9^-THC or its vehicle are presented in **Figure 3**. One-way ANOVA for the dose 0 mg/kg of Δ^9^-THC did not reveal any statistically significant effect, neither at the first postinjection [*F*(2,21) = 0.215, *p* = 0.809], nor at the second postinjection [*F*(2,21) = 0.903, *p* = 0.420). Paired sample *t*-test for the dose 0 mg/kg did not reveal any statistically significant difference between first and second postinjection session in any of the three groups [F0-VEH (*p* = 0.051), F0- Δ^9^-THC0.1 (*p* = 0.965), and F0-Δ^9^-THC1 (*p* = 0.874)]. One-way ANOVA for the dose 0.1 mg/kg of Δ^9^-THC, did not reveal any statistical significant effect in the asymptotic rate of responding of F1 of F0-Δ^9^-THC0.1 animals and F1 of F0-Δ^9^-THC1 animals neither at the first postinjection session [*F*(2,21) = 0.570, *p* = 0.574], nor at the second postinjection session [*F*(2,21) = 1.567, *p* = 0.232]. Paired sample *t*-test for the dose 0.1 mg/kg of Δ^9^-THC did not reveal any statistical significant difference between first and second postinjection session of the three groups [F0-VEH (*p* = 0.090), F0- Δ^9^-THC0.1 (*p* = 0.683), and F0-Δ^9^-THC1 (*p* = 0.682)]. Similarly, Δ^9^-THC at the dose of 0.5 mg/kg didn’t show any statistical significant effect in the asymptotic rate of responding of F1 animals neither at the first postinjection session [*F*(2,21) = 1.082, *p* = 0.357], nor at the second postinjection session [*F*(2,21) = 2.542, *p* = 0.103]. Paired sample *t*-test for the dose 0.5 mg/kg did not reveal any statistically significant difference between first and second postinjection session of the three groups [F0-VEH (*p* = 0.412), F0- Δ^9^-THC0.1 (*p* = 0.746), and F0-Δ^9^-THC1 (*p* = 0.343)]. One-way ANOVA for the dose 1 mg/kg of Δ^9^-THC did not reveal any statistical significant effect in the asymptotic rate of responding of F1 animals both at the first [*F*(2,21) = 0.050, *p* = 0.951] and the second postinjection session [*F*(2,21) = 2.121, *p* = 0.145]. No statistical significant effects were detected by using paired sample *t*-test between the first postinjection and second postinjection session in each of the three groups [F0-VEH (*p* = 0.107), F0- Δ^9^-THC0.1 (*p* = 0.488), and F0-Δ^9^-THC1 (*p* = 0.904)].

### Experiment 2: Effects of *d-*Amphetamine on Brain Stimulation Reward of F1 Animals

As expected, *d*-amphetamine at the dose of 0.5 and 1 mg/kg increased the rewarding efficacy of self-stimulation in drug naïve animals, i.e., F1 of F0-VEH animals (see **Figure 1B**) and caused leftward shifts in the rate-frequency function (see **Figure 1D**) Specifically, two-way ANOVA with repeated measures demonstrated a significant drug effect [*F*(3,21) = 169.862, *p* = 0.001] on the ICSS threshold. Paired sample *t-*test using Bonferroni’s adjustment for multiple comparisons revealed that *d*-amphetamine significantly decreased ICSS threshold at the doses of 0.5 (*p* = 0.001) and 1 mg/kg (*p* = 0.001). The dose of 0.1 mg/kg did not produce any significant effect (*p* = 0.331).

**FIGURE 4:**
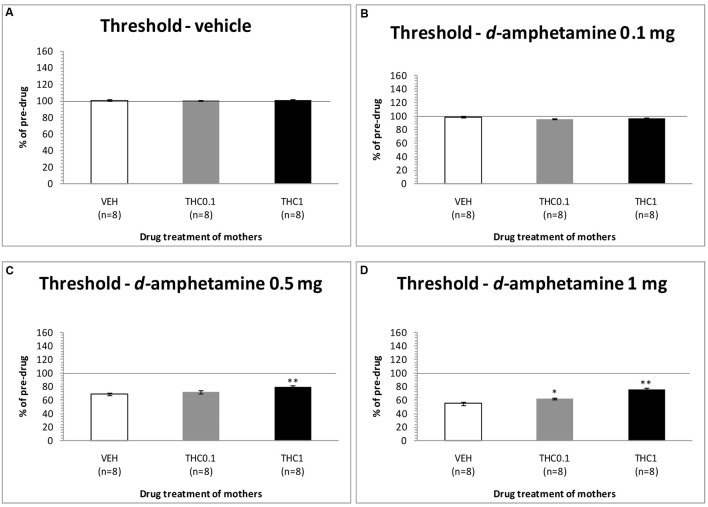
**Changes in self-stimulation threshold in F1 animals, expressed as percentage of predrug value, following acute i.p. administration of 0 mg/kg** **(A)**, 0.1 mg/kg **(B)**, 0.5 mg/kg **(C)**, and 1 mg/kg **(D)** of *d*-amphetamine. Horizontal axes represent the three groups of F1 animals. The *asterisk* (^∗^) signifies a statistically significant effect compared to the vehicle group (F1 of F0-VEH animals), ^∗^*p* < 0.05 and ^∗∗^*p* < 0.01.

The changes in ICSS threshold of F1 animals after acute systemic injection of *d*-amphetamine or its vehicle are presented in **Figure 4**. One-way ANOVA showed that the dose 0 mg/kg of *d*-amphetamine [*F*(2,21) = 0.027, *p* = 0.974] and the dose 0.1 mg/kg of *d*-amphetamine [*F*(2,21) = 2.048, *p* = 0.154] did not affect the threshold of F1 animals whose mothers had been exposed to Δ^9^-THC, during their adolescence, compared to F1 of F0-VEH animals. On the other hand, *d*-amphetamine at the dose 0.5 mg/kg significantly affected the threshold of F1 animals [*F*(2,21) = 6.275, *p* = 0.007]. Further analysis with independent sample *t*-test showed that *d*-amphetamine at the dose 0.5 mg/kg, increased the threshold for F1 of F0-Δ^9^-THC1 animals [*t*(14) = -3.528, *p* = 0.003], compared to F1 of F0-VEH group. However, this dose of *d*-amphetamine did not affect the threshold of F1 of F0-Δ^9^-THC0.1 animals [*t*(7) = -1.009, *p* = 0.330]. Finally, one-way ANOVA for the dose of 1 mg/kg of *d*-amphetamine revealed statistically significant effects [*F*(2,21) = 25.888, *p* = 0.001]. Analysis with independent sample *t*-test showed that *d*-amphetamine at the dose of 1 mg/kg increased the threshold of F1 of F0-Δ^9^-THC0.1 animals [*t*(14) = -2.848, *p* = 0.013]. Moreover, we found a greater increase in the threshold of F1 of F0-Δ^9^-THC1 animals [*t*(14) = -6.079, *p* = 0.001]. Rate-frequency functions (rate of lever pressing as a function of stimulation frequency) taken from representative F1 animals are presented in **Figure 6C** (mother treatment Δ^9^-THC 0.1 mg) and **Figure 6D** (mother treatment Δ^9^-THC 1 mg). These plots demonstrate that the reward-facilitating effect of *d*-amphetamine in F1 animals (vis-à-vis **Figure 1D**) has been diminished.

**FIGURE 5:**
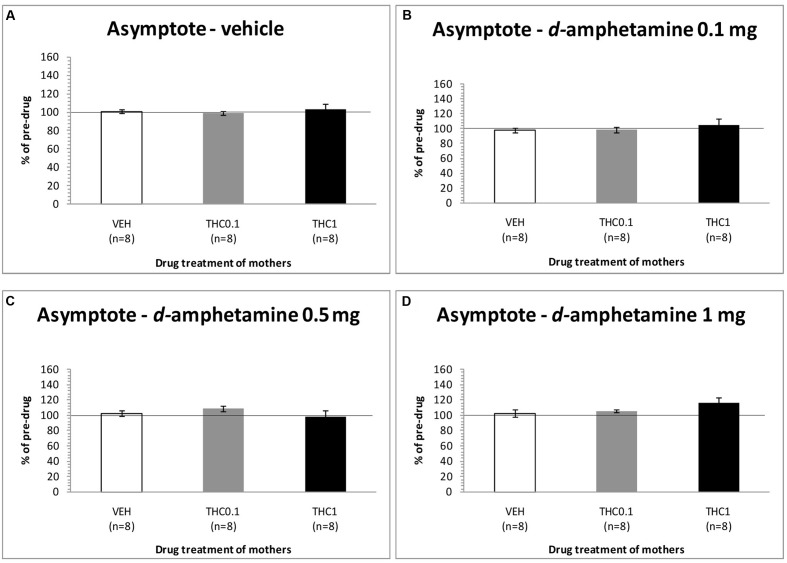
**Changes in asymptotic rate of responding in F1 animals, expressed as percentage of predrug value, following acute i.p. administration of 0 mg/kg** **(A)**, 0.1 mg/kg **(B)**, 0.5 mg/kg **(C)**, and 1 mg/kg **(D)** of *d*-amphetamine. Horizontal axes represent the three groups of F1 animals.

The changes in asymptotic rate of responding of F1 animals after acute systemic injection of *d*-amphetamine or its vehicle are presented in **Figure 5**. One-way ANOVA for the dose of 0 mg/kg of *d*-amphetamine did not reveal any statistical significant effect of this dose in the asymptotic rate of responding of F1 animals [*F*(2,21) = 0.190, *p* = 0.829]. Similarly, neither the dose of 0.1 mg/kg of *d*-amphetamine had any significant effect [*F*(2,21) = 0.614, *p* = 0.551]. Moreover, the dose of 0.5 mg/kg of *d*-amphetamine, did not affect the asymptotic rate of responding of F1 animals, which their mothers had been exposed to Δ^9^-THC [*F*(2,21) = 1.045, *p* = 0.369]. Finally, neither the dose 1 mg/kg of *d*-amphetamine had any statistical significant effect to the asymptotic rate of responding [*F*(2,21) = 1.747, *p* = 0.199].

**FIGURE 6:**
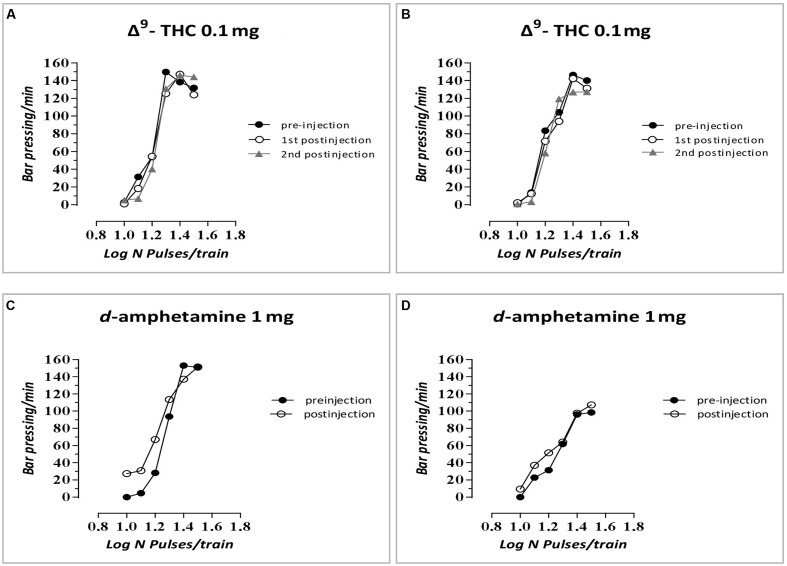
**Rate-frequency functions (rate of lever pressing as a function of stimulation frequency) taken from representative F1 animals.** Each plot represents data from a single animal under predrug and drug conditions. Rate frequency functions were obtained by logarithmically decreasing the frequency of the stimulation pulses from a value that sustained maximal lever pressing to one that failed to sustain lever pressing. **(A)** Mother treatment Δ^9^-THC 0.1 mg/F1 animal treatment Δ^9^-THC 0.1 mg, **(B)** mother treatment Δ^9^-THC 1 mg/F1 animal treatment Δ^9^-THC 0.1 mg, **(C)** mother treatment Δ^9^-THC 0.1 mg/F1 animal treatment *d*-amphetamine 1 mg, and **(D)** mother treatment Δ^9^-THC 1 mg/F1 animal treatment *d*-amphetamine 1 mg.

## Discussion

The current findings demonstrate transgenerational consequences of adolescent female cannabinoid exposure in the reward-modifying effects of drugs of abuse in the male offspring. Adolescence represents a critical phase in brain development (Spear, 2000). Importantly, during this period maturation of the major neurotransmitter systems is taking place. Thus, the adolescent brain is more vulnerable to the effects of addictive drugs, including cannabis. Several studies have examined the effects of periadolescent exposure to cannabinoids on brain function and behavior (Viveros et al., 2005; Schneider, 2008; Campolongo et al., 2011; Renard et al., 2014). While exposure to cannabinoids during pregnancy may alter offspring development and behavior, it remains a subject of active research whether cannabinoid exposure prior to pregnancy, and in particular during the adolescence period, can impact subsequent generations.

Thus, the results from the first experiment provide evidence that Δ^9^-THC at the dose of 0.1 mg/kg increased the ICSS threshold in F1 of F0-Δ^9^-THC0.1 animals and F1 of F0-Δ^9^-THC1 animals. In other words, the dose of 0.1 mg/kg of Δ^9^-THC that consistently decreases the ICSS threshold (Katsidoni et al., 2013) induced anhedonic effects, i.e., increased the ICSS threshold in F1 male animals whose mothers were exposed during their adolescence to Δ^9^-THC (0.1 or 1 mg/kg). This anhedonic effect was demonstrated by the rightward shifts of the rate-frequency curve (see **Figure 1C** vis-à-vis **Figures 6A,B**). The doses we selected were based on our previous study showing biphasic effects of Δ^9^-THC on ICSS, i.e., a low dose of Δ^9^-THC (0.1 mg/kg) decreased ICSS thresholds, whereas a higher dose (1 mg/kg) increased ICSS thresholds (Katsidoni et al., 2013). In the present experiment, the anhedonic effects of the 1 mg/kg dose of Δ^9^-THC remained unaltered by adolescent exposure of the F0 female rats to Δ^9^-THC. Notably, adolescent female exposure to either 0.1 or 1 mg/kg Δ^9^-THC that under acute conditions, either decrease or increase ICSS thresholds (Katsidoni et al., 2013; see also **Figure 1A**), elicited unidirectional changes in the Δ^9^-THC ICSS thresholds in the male offspring (see **Figure 2**).

The results of the second experiment provide evidence that administration of the two highest doses of *d*-amphetamine, namely 0.5 and 1 mg/kg, produced a smaller decrease in the ICSS threshold of F1 animals whose mothers were exposed to Δ^9^-THC during adolescence compared to control animals, decreasing, thus, the reward-facilitating effects of *d*-amphetamine. Interestingly, the increased ICSS threshold was greater in the F1 animals whose mothers were exposed to 1 mg/kg Δ^9^-THC, rather than in the F1 animals whose mothers were exposed to 0.1 mg/kg Δ^9^-THC during adolescence (see **Figure 4**).

The present experiments (see **Figure 1**) replicated our previews findings that a low dose of Δ^9^-THC (0.1 mg/kg) administered acutely in drug naïve animals increases the rewarding efficacy of ICSS, whereas a high dose (1 mg/kg) induces anhedonic effects (Katsidoni et al., 2013). Similarly, the reward-facilitating effect of *d*-amphetamine observed in the present study is in agreement with several previous reports utilizing acute regimens (Esposito et al., 1980; Mavrikaki et al., 2010; Bauer et al., 2013a,b). The fact that the reward-facilitating effects of the 0.1 mg/kg dose of Δ^9^-THC and the 0.5 and 1.0 mg/kg doses of *d*-amphetamine were abolished and diminished, respectively, in the F1 offspring of females that were exposed to Δ^9^-THC during their puberty, suggests that significant transgenerational effects may arise in the male offspring of mothers which are exposed to Δ^9^-THC during their adolescence. We could speculate that the exposure of F0 female animals to Δ^9^-THC (0.1 and 1 mg/kg) during their adolescence, resulted in epigenetic changes, which were inherited by their descendants through the germline (Skinner, 2011). Thus, when the F1 of F0-Δ^9^-THC animals received the dose 0.1 mg/kg of Δ^9^-THC, or the 0.5 and 1.0 mg/kg doses of *d*-amphetamine, these particular transgenerational effects were revealed as a decrease of their reward-facilitating effects, as observed in our ICSS studies. Interestingly, no significant differences were observed for the reward-blocking doses of 0.5 and 1 mg/kg of Δ^9^-THC between the different groups. According to our results, the different groups of animals did not present any statistical significant differences in the maximum rate of responding, and chronic administration of Δ^9^-THC during adolescence in female rats did not affect significantly the asymptotic rate of responding of male offspring (see **Figures 3**, **5**). Thus, the changes in ICSS thresholds observed after Δ^9^-THC or *d*-amphetamine injection were not confounded by performance effects, and the effects of Δ^9^-THC and *d*-amphetamine on ICSS thresholds cannot be attributed to effects on motor activity. The failure to observe transgenerational effects on the asymptotic rate of responding suggests that the drug exposure of mothers during their adolescence did not affect the performance of their male offspring on lever pressing. This is also consistent with previous reports on homologous findings with acute administration of cannabinoids and *d*-amphetamine in drug naïve animals (Vlachou et al., 2003, 2005, 2007; Mavrikaki et al., 2010; Katsidoni et al., 2013).

As the F1 offspring of female rats that were exposed to Δ^9^-THC during their adolescence exhibit a phenotype of diminished reward-facilitating effects of Δ^9^-THC and *d*-amphetamine, it is unclear what led to the appearance of this phenotype and if other behavioral variables have some relevance. Interestingly, chronic exposure to Δ^9^-THC during adolescence may affect the behavioral repertoire of experimental animals. For example, Zamberletti et al. (2012) identified gender-dependent behavioral effects of adolescent Δ^9^-THC exposure in adult rats, such as a decrease in recognition memory or an increased passive coping strategy; such effects may affect maternal behavior and lead to epigenetic alterations.

The mechanism underlying the occurrence of these transgenerational effects remains unknown. It is possible that neuroadaptive changes induced to the mothers of the F1 animals by the chronic adolescent exposure to Δ^9^-THC may be responsible for the observed effects. Previous work has shown that the dopaminergic neurons in the ventral tegmental area of animals pretreated during their adolescence with Δ^9^-THC were significantly less responsive to the stimulatory action of the cannabinoid agonist WIN55,212-2 and Δ^9^-THC-exposed rats displayed a reduced capacity for WIN55,212-2 to increase dopamine levels in the nucleus accumbens shell (Scherma et al., 2016). Similar results have been observed after adolescent exposure to the cannabinoid agonist WIN55,212-2 (Pistis et al., 2004). Theoretically, changes in the mesolimbic dopaminergic system and its limbic neuronal sequelae in response to Δ^9^-THC exposure in adolescent female results, may result in a diminution of the reward-facilitating effect of Δ^9^-THC and *d*-amphetamine in theirs adult male offspring observed in the present study.

A recent study that examined the transgenerational effects of cocaine also provided evidence for behavioral and neurobiological alterations in the offspring (Vassoler et al., 2013). Specifically, rats whose fathers were exposed to cocaine during their puberty, showed delayed acquisition of cocaine self-administration. The authors claim that the reduction in the reinforcing effects of cocaine is due to the cocaine that the male animals received during their puberty, which through an epigenetic mechanism cause reprograming of the germline of F1 offspring, leading in this way their descendants to inherit a cocaine-resistant phenotype. Moreover, in another major study, rats whose mothers were exposed to morphine showed decreased morphine self-administration and attenuated relapse-like behavior; in addition, genes related with neurodevelopment and synaptic plasticity were dysregulated within the nucleus accumbens of the affected rats (Vassoler et al., 2017). These behavioral data are similar with those reported in our study. Thus, Δ^9^-THC to which female rats were exposed during their adolescence, through an unknown epigenetic mechanism, may have caused reprograming of the germline of these animals leading in this way their offspring to inherit and present diminished reinforcing efficacy of Δ^9^-THC and *d*-amphetamine.

Interestingly, several studies have shown that cannabinoids interact with other commonly used and abused drugs, such as psychostimulants; also, the endocannabinoid system regulates the reward-facilitating effects of psychostimulants, including *d*-amphetamine and cocaine. For example, previous studies from our group have shown that the cannabinoid receptor agonist WIN55,212-2 as well as the endocannabinoid neurotransmission enhancer AM-404 attenuated the reward-facilitating effect of cocaine in drug naïve rats (Vlachou et al., 2003; Vlachou et al., 2008). Similarly, WIN55,212-2 decreases intravenous cocaine self-administration in rats (Fattore et al., 1999).

## Conclusion

Our study provides evidence that adolescent female exposure to Δ^9^-THC can change the phenotype in the subsequent generation of male animals, preventing and attenuating the well-established, reward-facilitating effects of Δ^9^-THC (0.1 mg/kg) and *d*-amphetamine (0.5 and 1 mg/kg), respectively. These transgenerational effects occur in the absence of *in utero* exposure and provide evidence for transgenerational effects of adolescent female drug exposure on a subsequent generation, even in the absence of direct drug exposure during pregnancy. The observed diminished reward-facilitating effect of Δ^9^-THC and *d*-amphetamine does not necessarily imply a “protective” phenotype for the offspring in regard to addiction liability. Alternatively, these effects may reflect an anhedonic phenotype with lower sensitivity to natural rewards and susceptibility to addictive behaviors. This hypothesis is also supported by the studies of the Byrnes group (Vassoler et al., 2013, 2017). An important consideration for future studies is the elucidation of the specific mechanism(s) underlying these effects, given their impact on understanding offspring maladaptive behavior after adolescent female exposure to cannabinoids.

## Author Contributions

Conceived and designed the experiments: GPa. Performed the experiments and analyzed the data: GPi and DS. Wrote the paper: GPa, GPi, DS, and GN.

## Conflict of Interest Statement

The authors declare that the research was conducted in the absence of any commercial or financial relationships that could be construed as a potential conflict of interest.
